# Impact of Caudate Lobe Resection on Overall Survival and Liver Disease-Free Survival in Colorectal Liver Metastases: A Pilot Study

**DOI:** 10.5152/tjg.2025.24669

**Published:** 2025-01-27

**Authors:** Melih Can Gül, Emin Demirel

**Affiliations:** 1 Department of Gastroenterology Surgery, Afyonkarahisar State Hospital, Türkiye; 2 Department of Radiology, Afyonkarahisar Health Sciences University, Türkiye

**Keywords:** Colorectal carcinoma, liver, metastasis

## Abstract

**Background/Aims:**

The objective of this study was to evaluate the impact of the resected caudate lobe on survival, particularly in the context of anatomical resection of liver metastases in colorectal cancers without metastases in the caudate lobe.

**Materials and Methods:**

Patient data were extracted from the dataset titled “Preoperative CT and Survival Data for Patients Undergoing Resection of Colorectal Liver Metastases (CRLM).” The analysis specifically concentrated on individuals who underwent complete caudate lobe resection in the absence of radiological signs of metastasis within the caudate lobe itself. To discern the distinct impact of caudate lobe resection on patient outcomes, propensity score matching (PSM) was applied to control for variations across other relevant clinical parameters. Overall survival (OS) and liver disease-free survival (liver DFS) were calculated using the Kaplan–Meier method, while the log-rank test was used to compare survival outcomes between groups.

**Results:**

The analysis revealed that patients who underwent total caudate lobe resection exhibited significantly improved OS rates, both in the complete dataset and following PSM (*P* < .001, HR: 0.43, 95% CI: 0.26-0.72; *P* = .024, HR: 0.65, 95% CI: 0.59-0.79, respectively). Additionally, liver DFS outcomes were found to be superior in patients who had caudate lobe resection, in both the full dataset and the propensity-matched cohort (*P* = .014, HR = 0.46, 95% CI: 0.24-0.85; *P* = .026, HR = 0.5, 95% CI: 0.37-0.79, respectively).

**Conclusion:**

These findings suggest that incorporating total caudate lobe resection into the surgical management of CRLM may offer substantial benefits in terms of both OS and liver-specific disease-free survival.

**Main Points**

- The principal aim of this study is to ascertain the effect of incorporating a complete caudate lobe resection on overall survival (OS) and liver disease-free survival (liver DFS) among patients who have undergone anatomical resection to address metastases associated with lobar portal vascular structures, particularly in instances where no radiological evidence of macrometastasis is present within the caudate lobe itself.
- The study was prompted by the possibility that the caudate lobe may serve as a reservoir for micrometastases due to its intricate vascular and biliary anatomy, particularly in the context of hilar cholangiocarcinomas.
- Propensity score matching revealed that total caudate resection markedly enhanced OS and liver DFS compared to patients who did not undergo such resection.
- The findings suggested that caudate lobectomy confers superior oncological outcomes in terms of OS and DFS, even in the absence of caudate lobe metastases.

## Introduction

Colorectal cancer (CRC) represents a significant global health burden, ranking as the second most frequently diagnosed cancer in women and the third in men. This malignancy accounts for approximately 10% of all newly diagnosed cancer cases and cancer-related mortalities annually, thereby underscoring its considerable impact on public health worldwide.^1^

Among visceral organs, the liver is the primary site for hematogenous metastasis and the most frequent location for metastatic spread in individuals with CRC. Over 25% of CRC patients develop colorectal liver metastases (CRLM) at some stage during their disease progression, which highlights the liver’s critical role in the metastatic pattern of CRC.^2^ The management of CRLM has consequently become a complex, interdisciplinary domain that integrates expertise across various fields, including radiology (which encompasses cross-sectional imaging, nuclear medicine, and interventional radiology), oncology, liver surgery, colorectal surgery, and pathology. In order to provide optimal patient care within this context, it is necessary to conduct comprehensive clinical, radiological, and biomarker evaluations. Despite the limited evidence available for this highly heterogeneous patient population, the central objective remains the maximization of CRLM resection through every feasible approach, as this provides the greatest potential for long-term survival and a possible cure. However, even with the use of systemic chemotherapy, intrahepatic recurrence following curative surgery remains common, with an estimated two-thirds of patients experiencing a recurrence within 3 years.^3^

Recently, there has been a shift toward parenchyma-sparing or non-anatomic resections over anatomic resections (AR) in CRLM patients to conserve liver parenchyma. As surgeons have become more receptive to narrower surgical margins in CRLM resections, this approach has promoted the preservation of liver parenchyma, enabling a greater remnant liver volume.^4^

Proponents of AR in the context of CRLM emphasize the importance of considering individual tumor biology in surgical planning. Somatic mutations in the RAS gene have been correlated with worse long-term outcomes in metastatic CRC patients. Moreover, these mutations have been linked to the surgical margins achieved after hepatectomy. Patients with RAS-mutant CRLM tend to exhibit narrower surgical margins post-hepatectomy. RAS-mutant tumors are known to have a higher likelihood of microscopic invasion into the intrahepatic vascular and biliary systems, potentially leading to latent micrometastases along the portal triad. This has led researchers to suggest the possible advantage of AR in managing RAS-mutant CRLM.^5^^,^^6^

The caudate lobe of the liver was the subject of scientific study as early as the 13th century. This anatomically distinct lobe is situated deep within the dorsal segment of the liver, positioned between the inferior vena cava (IVC) and the portal triad. The caudate lobe’s distinctive anatomical position and intricate venous drainage system, which draws blood from both the primary portal veins and the biliary system,^7^ have rendered it a topic of considerable scientific and clinical discourse for an extended period. A comprehensive meta-analysis has demonstrated that the incorporation of either total or partial resection of the caudate lobe in the surgical management of hilar cholangiocarcinoma can result in significant improvements in both overall and disease-free survival (DFS). In such cases, the identification of micrometastases within the biliary tract has been identified as a crucial factor contributing to enhanced survival outcomes.^8^^,^^9^

The primary objective of the present study was to evaluate the impact of caudate lobe resection on overall survival (OS) and liver-specific disease-free survival (DFS) (liver DFS) in patients with CRLM involving both hepatic lobes. By assessing these outcomes, this research aims to ascertain the potential survival benefit of caudate lobe resection in the context of bilobar CRLM management. Considering the complex anatomy of this region—which may act as a potential reservoir for occult metastases—we aimed to determine whether the inclusion of caudate lobe resection would influence survival outcomes. Given the complex anatomical structure of this region, which may serve as a potential reservoir for occult metastatic cells, we employed propensity score matching (PSM) to increase the accuracy and reliability of our results.

## Materials and Methods

### Patient Selection

The dataset, which comprises data on 197 patients diagnosed with CRLM, includes both preoperative and postoperative CT imaging and survival information specifically for individuals who underwent surgical resection of CRLM.^10^^,^^11^ The dataset was accessed via the cancer imaging archive (TCIA).^12^ Additionally, detailed patient characteristics, including age, sex, body mass index (BMI), carcinoembryonic antigen (CEA) levels, maximum tumor dimensions, the presence of synchronous CRLM, node-positive primary status, the occurrence of multiple metastases, bilobar disease, extrahepatic disease, and major comorbid conditions, were also retrieved from TCIA. As the TCIA database lacks any information that could be used to identify the patients personally, obtaining informed consent was deemed unnecessary for this analysis.

In order to be included in this study, patients were required to have undergone contrast-enhanced computed tomography (CT) imaging captured in both the preoperative and postoperative portal venous phases. This ensured a consistent basis for evaluating hepatic and vascular structures before and after surgical intervention. Patients were excluded from the study if they met any of the following criteria: (a) the presence of metastases situated within 1 cm of the caudate lobe or directly involving the caudate lobe itself; (b) imaging studies that were either of inadequate quality or incomplete, rendering them unsuitable for accurate analysis; and (c) the absence of available survival data, which precluded inclusion in the survival analysis.

The definition of total caudate lobe resection was established to encompass the complete removal of both the Spiegelian lobe and the paracaval region. In cases where preoperative imaging studies were thoroughly evaluated, total caudate lobe resection was commonly conducted in conjunction with anatomical resection of either the right or left hepatic lobe, particularly in instances where there was significant tumor involvement of the major portal structures in the respective lobes. The completeness of the caudate lobe resection was meticulously verified by comparing preoperative and postoperative tomography scans. This evaluation was conducted by a general surgeon with a decade of experience and a radiologist with over 10 years of expertise in the field. In instances where the resection status was uncertain, as with 2 particular patients, a secondary evaluation was performed by an additional radiologist with 20 years of experience. Following this, a unanimous decision regarding the resection status was reached.

### Data Availability Statement

While the current study includes data from human clinical trials, the data analyzed were obtained from the cancer imaging archive (TCIA) (https://doi.org/10.7937/QXK2-QG03), which has been previously archived and made publicly available and does not impose privacy or ethical restrictions. No changes were made to the existing dataset. Therefore, no additional permission was required from the local ethics committees where the authors work.

### Statistical Analysis

Statistical analysis was performed using SPSS version 25 (IBM SPSS Corp.; Armonk, NY, USA). Continuous variables were given as (mean ± standard deviation), and categorical variables were given as number (ratio). Normality tests for continuous variables were conducted using the Kolmogorov–Smirnov and Shapiro–Wilk tests. Comparisons between groups were made using the following statistical tests: chi-square test for categorical variables, Student’s *t*-test for normally distributed continuous variables, and Mann–Whitney *U* test for non-normally distributed continuous variables.

We also used PSM with a 1 : 1 ratio to minimize selection bias and adjust the imbalance between groups. SPSS R plug-in (SPSS R Essentials) was applied for matching.^13^ We used the SPSS “PS Matching” feature to perform propensity score-matched analysis. Matching factors include age, gender, BMI, CEA, maximum tumor size, synchronous CRLM, node-positive primary, multiple metastases, bilobar disease, extrahepatic disease, and majör comorbidity. Patients with caudate lobe resected/non resected were matched 1 : 1 in a multivariable logistic analysis using stepwise regression based on a greedy matching algorithm with a caliper of 0.05 times the standard deviation (SD) of the logit. After applying 1 : 1 PSM, 23 eligible patients were matched to each group.

The Kaplan–Meier curve was used to calculate the OS and liver DFS curve, and the log-rank test was applied to investigate differences in OS and liver DFS between caudate lobe resected non-resected groups. *P* values < .05 were considered to indicate statistical significance.

## Results

Demographic data of the overall dataset is shared in Table 1. In the evaluation made according to all parameters, no significant difference was detected in the patient groups with and without caudate lobe resection.

A comprehensive analysis was conducted on the dataset to ascertain the prevalence of local liver recurrence and distant metastases involving the lungs, bones, and lymph nodes. The incidence of metastatic spread to distant organs, specifically the lungs, bones, or lymph nodes, was not statistically significantly different between the cohort of patients who underwent complete caudate lobe resection and those who did not. This indicates that caudate resection did not significantly impact the rate of metastasis to these sites. This finding suggests that caudate resection did not significantly impact the incidence of metastasis to these distant sites.

In examining OS and liver metastasis recurrence following resection, patients who received total caudate lobe resection demonstrated superior OS and DFS outcomes compared to those who did not undergo this procedure. This was observed in both the entire patient cohort and the subgroup analyzed through propensity score matching.

**Figure 1.**
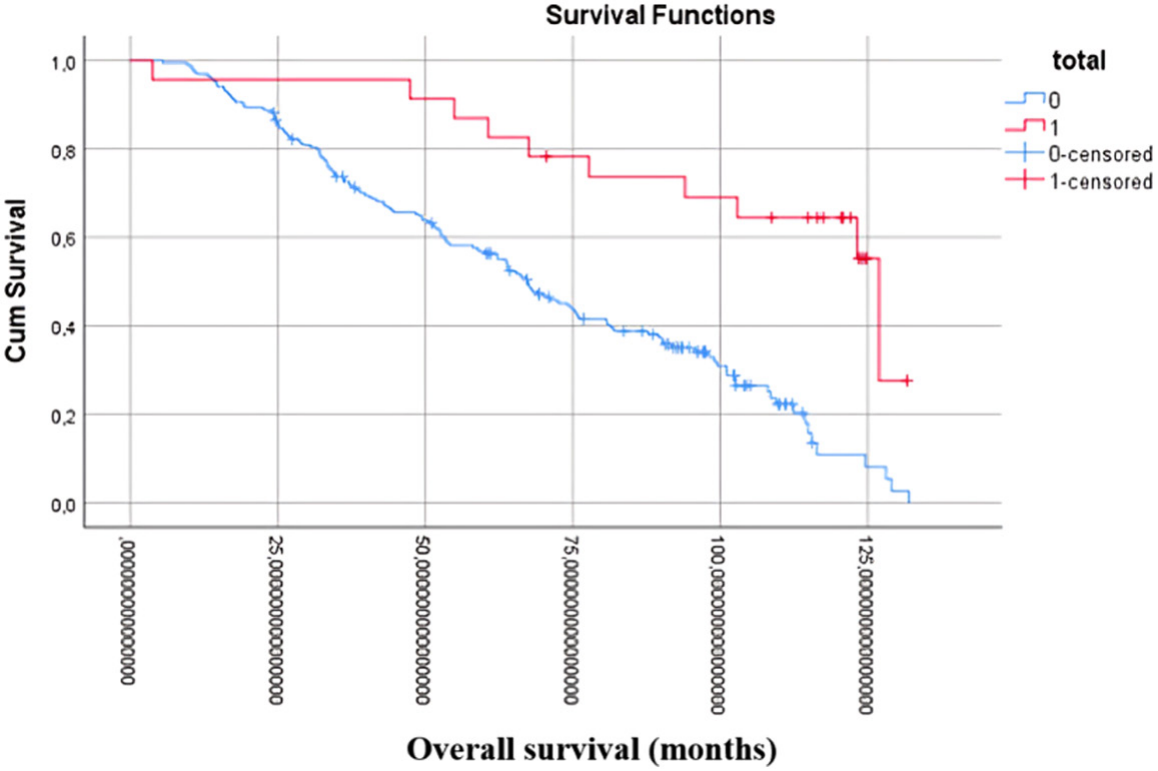
Kaplan–Meier curve for OS in the whole case set.

**Figure 2.**
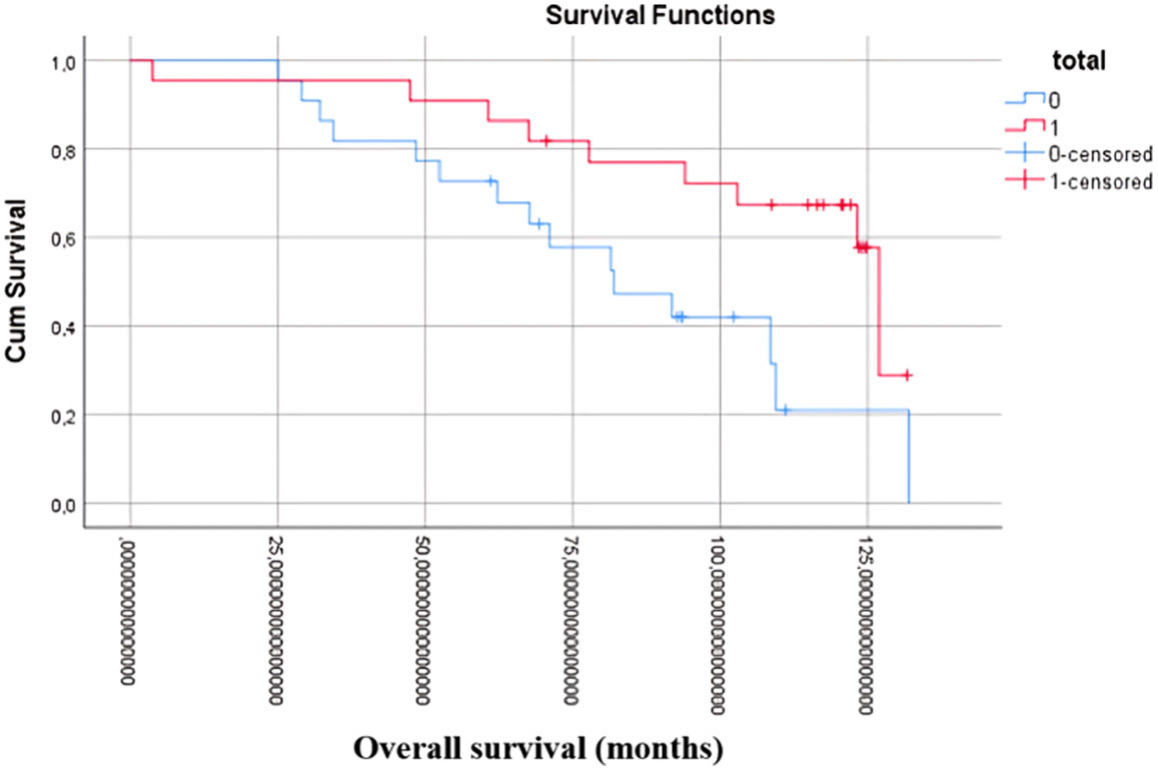
Kaplan–Meier curve OS in case set after PSM.

The dataset further revealed a correlation between total caudate lobe resection and improved OS, as illustrated in Figure 1 (*P* < .001), (HR: 0.43; 95% CI: 0.26-0.72) and Figure 2 (log-rank test, *P* = .024), (HR = 0.65; 95% CI: 0.59-0.79).

**Figure 3.**
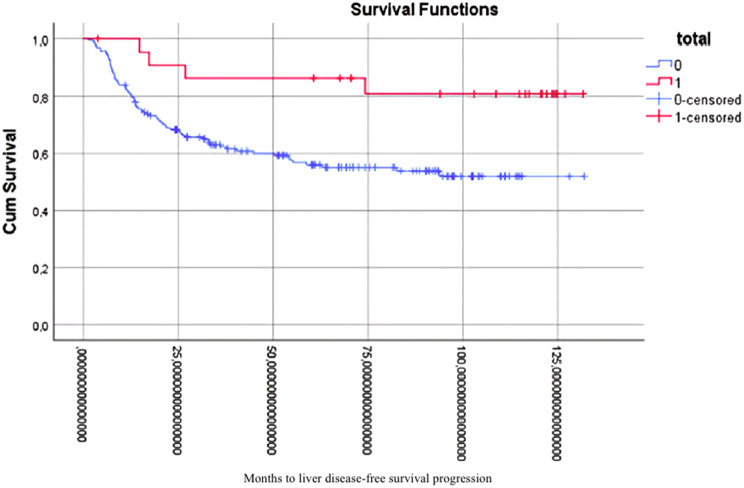
Kaplan–Meier liver disease-free survival in the whole case set.

**Figure 4.**
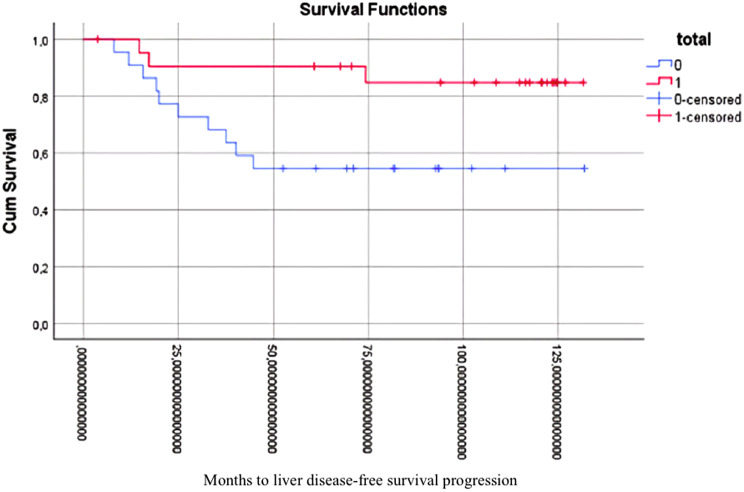
Kaplan–Meier liver disease-free survival in case set after PSM.

Furthermore, patients who underwent complete caudate lobe resection demonstrated enhanced outcomes with respect to liver recurrence-free survival. The comprehensive dataset indicated enhanced survival rates, as demonstrated in Figure 3 (log-rank test, *P* = .014; HR: 0.46; 95% CI: 0.24-0.85). Further analysis revealed that patients who underwent total caudate lobe resection following the application of PSM exhibited significantly enhanced liver DFS, as illustrated in Figure 4 (log-rank test, *P* = .026; HR: 0.51; 95% CI: 0.37-0.79).

## Discussion

Colorectal cancer is acknowledged as the second leading cause of cancer-related mortality globally, a ranking that becomes even more pronounced when cases involving metastatic progression are taken into account.^14^ Among the various organs susceptible to CRC metastasis, the liver is the most frequently affected, with approximately 25% of patients presenting with hepatic metastatic involvement at the time of their initial diagnosis. Currently, hepatectomy remains the most effective therapeutic intervention for managing colorectal metastases, contingent upon the location of the metastasis and the adequacy of residual liver volume to support safe resection and ensure optimal postoperative liver function.^15^

Nevertheless, there exists a notable scarcity of research specifically investigating how the precise anatomical location of hepatic metastases might influence patient prognosis. This indicates a gap in the existing literature that warrants further exploration to fully understand the implications of metastatic positioning within the liver. Kuo et al^16^ propose that liver metastases situated in the central segments and caudate lobe may portend a poor prognosis following hepatectomy and are associated with early recurrence in CRC.

The caudate lobe was initially the subject of study as early as the 13th century. Thereafter, its anatomical structure underwent further refinement as the delineation of modern caudate segments advanced our understanding of this unique hepatic region.^17^ The caudate lobe receives its blood supply from the portal venous system via 3 distinct pathways: specifically, the right and left branches of the portal vein, as well as the main trunk of the portal vein itself. This vascular configuration serves to illustrate the intricate and complex nature of the blood supply network.^18^ Despite the availability of non-surgical therapies, including ablation techniques and transcatheter arterial embolization, for the management of tumors within the caudate lobe, these approaches are typically regarded as less effective than surgical resection in achieving comprehensive tumor control. Consequently, surgical resection is widely regarded as the primary and most effective therapeutic option for neoplasms situated within this lobe.^19^ Additionally, the bile duct associated with the caudate lobe is in close proximity to the common bile duct at the hepatic hilum and is almost invariably involved in cases of perihilar cholangiocarcinoma. Therefore, resection of the caudate lobe is often included as a standard component in the surgical treatment plan for this malignancy.^20^

In many studies investigating the metastasis distribution of CRCs according to the Couinaud segmental classification of the liver, the caudate lobe was excluded from the study because it was known to have isolated portal, biliary tract, and vena cava drainage and inter-segmental homogenization could not be achieved during the study.^21^

In patients diagnosed with CRC, those presenting with metastasis in the caudate lobe, whether as an isolated occurrence or in conjunction with metastasis in another hepatic segment, exhibited a lower survival rate compared to patients without caudate lobe involvement.^22^

The involvement of CRLMs within the caudate lobe introduces a significant degree of complexity to the undertaking of a curative hepatectomy, primarily due to the challenging anatomical location of this lobe. The caudate lobe is situated in a confined and anatomically intricate space, in close proximity to major vascular structures such as the IVC, the portal vein confluence, and the junction where the left and middle hepatic veins converge. This positioning highlights the caudate lobe’s pivotal role and potential implications in surgical procedures, particularly in the context of oncological liver resections.^23^

The mobilization of the caudate lobe from the superior vena cava through the ligation of short hepatic veins during resection is often associated with an increased risk of intraoperative bleeding and associated morbidity, particularly among surgeons with limited experience. However, recent studies indicate that as surgical expertise has advanced, the frequency of caudate lobe resections has increased in parallel. It is noteworthy that, despite the inclusion of patients who underwent vascular resection, these studies have not demonstrated significant differences in postoperative complications between groups.^24^

A comprehensive review of the existing literature on caudate lobe resection in the surgical management of hepatic metastases originating from colorectal carcinoma reveals that the patients included in prior studies typically presented with either multiple metastases involving the caudate lobe or isolated metastases confined to this lobe.^25^

In this context, our study stands as the first to perform a comparative analysis of OS, DFS, and distant organ metastasis outcomes between 2 distinct groups of patients: those who underwent complete caudate lobe resection as part of their liver resection in cases without metastases in the caudate lobe (specifically, the segment closest to the portal vein and the first segment to drain into it) and those who did not undergo caudate resection.

In a related study evaluating the outcomes of extended left hepatectomy combined with caudate lobe resection in CRC patients with metastases in both the left and caudate lobes, 17 patients underwent a combination of left hepatectomy and caudate lobectomy. In addition, a separate cohort of 14 patients underwent extended left hepatectomy without caudate resection, with analysis showing comparable rates of postoperative liver recurrence and morbidity between these groups. However, postoperative distant organ metastases were more commonly observed in the caudate lobectomy group due to caudate lobe metastases, a finding likely due to the significant caval drainage associated with the caudate lobe.^26^

Our data series showed no significant difference in the incidence of distant organ metastases between patients with and without caudate resection. However, postoperative liver recurrence was significantly higher in the parenchyma-sparing group that did not undergo caudate resection, showing a statistically significant difference.

The optimal extent of hepatectomy required for the treatment of CRC liver metastases remains a subject of ongoing debate in the medical community.^27^ Recent meta-analyses comparing parenchyma-sparing surgery with anatomical resection have found no significant differences in OS rates at 1, 3, and 5 years between these groups. However, parenchyma-sparing surgery has been associated with lower success rates in achieving negative resection margins, higher intrahepatic recurrence rates, and a greater need for repeat hepatectomy.^4^^,^^28^

As a matter of fact, in our study, the patient group in which the caudate lobe was included in the resection was found to be superior in terms of OS and liver DFS. We do not have a definite idea as to why patients with caudate lobe resection had superior DFS and OS. However, we think that the genotype structure of the primary tumor may be a factor in this. In many retrospective cohorts, it has been reported that parenchyma-sparing surgery is more disadvantageous in terms of OS and DFS in the liver in patient groups with RAS mutation.^29^ It has been reported that the presence of undetectable micrometastases in the liver in patients with KRAS mutation predisposes them to R1 resection with parenchymal preservation and to reduced OS in patients with tumor recurrence in the residual liver.^30^

In our study, we think that the caudate lobe may be a micrometastasis bed. Although OS and liver DFS were found to be more favorable in caudate lobectomy groups with higher parenchymal resection in our study, the presence of mutations in the parenchymal sparing group is not known due to the lack of genome data in the data set.

Despite a reduction in the number of patients following PSM, the data from the study indicate that capsular or subcapsular-related main portal lesions that can be excised via non-anatomical resections should not be combined with the caudate lobe in cases where the metastases are not associated with arterial branches. Our findings indicate that the inclusion of the caudate lobe in the resection may enhance survival outcomes in patients who are eligible for anatomical major hepatectomy due to significant vascular invasion without caudate lobe involvement or with multiple metastases confined to a single lobe, with at least 1 cm distance from the caudate lobe.

There are very important limitations in our study. Due to the retrospective planning of the study, the necessary optimizations could not be made. The effect of isolated caudate lobe resection on patients could not be investigated. In fact, the group examined included extended right and left lobe resections. Given the limited number of patients, it was not possible to analyze the disease in each lobe separately. For this reason, no recommendation has been made to add caudectomy to a specific group. The genomic profile of the primary tumor is unknown. Our article was written on an open access dataset, and as we were not able to access all the perioperative and early postoperative morbidity and mortality data of the patients, no comparison could be made between the groups in terms of complications and early mortality. Therefore, the groups compared are quite heterogeneous. For a very common disease like CRLM, the sample group is relatively small. For all these reasons, our study is actually a pilot study.

In conclusion, we hypothesized that the caudate lobe is more susceptible to micrometastases because it is an isolated, specific lobe with high vascular and biliary drainage located at the hilar junction. Although superior oncological outcomes in terms of OS and DFS were found in the surgery group in which resection was added, the effect of these on the disease is not known since the genomic status of the patients, tumor biology, and tumour differentiation were not revealed due to the insufficiency of the current data set. Nevertheless, our findings are promising.

## Figures and Tables

**Table 1.** Demographic Data of Whole Datasets

	No Caudate Lobe Resection Added (n: 169)	Added Caudate Lobe Resection (n: 23)	*P*
**Age** (mean (SD))	59.36 (12.23)	63.17 (11.62)	.373
**Body mass index** (mean (SD))	27.27 (4.92)	27.90 (4.81)	.841
**Clinic risk score** (mean (SD))	2.05 (0.95)	1.73 (0.92)	.762
**Carcinoembryonic antigen** (mean (SD))	30.56 (96.16)	21.77 (34.48)	.730
**Maximum tumor size** (mean (SD))	3.51 (2.63)	3.30 (2.21)	.491
**Overall survival (months)** (mean (SD))	62.99 (33.14)	99 (33.6)	.762
**Sex** (male n, %)	102 (60.4)	12 (52.2)	.454
**Major comorbidity** (n, %)	93 (55)	13 (56.5)	.893
**Node positive primary** (n, %)	58 (34.3)	9 (39.1)	.650
**Synchronous CRLM** (n, %)	95 (56.2)	13 (56.5)	.978
**Multiple metastases** (n, %)	99 (58.6)	13 (56.5)	.851
**Bilobar disease** (n, %)	74 (43.8)	10 (43.5)	.978
**Extrahepatic disease** (n, %)	12 (7.1)	4 (17.4)	.094

SD: Standart deviation; CRLM: Colorectal cancer liver metastasis
